# CRP under 130 mg/L rules out the diagnosis of *Legionella pneumophila* serogroup 1 (URINELLA Study)

**DOI:** 10.1007/s10096-024-04814-x

**Published:** 2024-03-26

**Authors:** Timothée Klopfenstein, Souheil Zayet, Samantha Poloni, Vincent Gendrin, Damien Fournier, Jean-Baptiste Vuillemenot, Philippe Selles, Alain Dussaucy, Gaelle Coureau, Marta Avalos-Fernandez, Lynda Toko, Pierre-Yves Royer, Charles-Eric Lavoignet, Bouchra Amari, Marc Puyraveau, Catherine Chirouze

**Affiliations:** 1 Infectious Diseases and Tropical Department, Nord Franche-Comté Hospital, 90400 Trevenans, France; 2 Infectious Diseases and Tropical Department, Besançon University Hospital, Besançon, France; 3 Bacteriology Laboratory, Besançon University Hospital, Besançon, France; 4 Bacteriology Laboratory, Nord Franche-Comté Hospital, Trevenans, France; 5 Medical Information Department, Nord Franche-Comté Hospital, Trevenans, France; 6 Medical Information Department, Besançon University Hospital, Besançon, France; 7 University of Bordeaux, Population Health Research Center, UMR U1219, INSERM, Bordeaux, France; 8 Emergency Department, Nord Franche-Comté Hospital, Trevenans, France; 9 Methodology Unit, Clinical Investigation Center INSERM 1431, Besançon University Hospital, Besançon, France; 10 UMR-CNRS 6249 Chrono-Environnement, Department of Infectious and Tropical Diseases, Université de Franche-Comté, CHU Besançon, 25000 Besançon, France

**Keywords:** Legionnaire’s disease, Legionellosis, Urinary Antigen Test, C-reactive protein, Negative Predictive Value

## Abstract

**Introduction:**

In case of pneumonia, some biological findings are suggestive for Legionnaire’s disease (LD) including C-reactive protein (CRP). A low level of CRP is predictive for negative *Legionella* Urinary-Antigen-Test (L-UAT).

**Method:**

Observational retrospective study in *Nord-Franche‐Comté* Hospital with external validation in *Besançon* University Hospital, France which included all adults with L-UAT performed during January 2018 to December 2022. The objective was to determine CRP optimal threshold to predict a L-UAT negative result.

**Results:**

URINELLA included 5051 patients (83 with positive L-UAT). CRP optimal threshold was 131.9 mg/L, with a negative predictive value (NPV) at 100%, sensitivity at 100% and specificity at 58.0%. The AUC of the ROC-Curve was at 88.7% (95% CI, 86.3–91.1). External validation in *Besançon* Hospital patients showed an AUC at 89.8% (95% CI, 85.5–94.1) and NPV, sensitivity and specificity was respectively 99.9%, 97.6% and 59.1% for a CRP threshold at 131.9 mg/L; after exclusion of immunosuppressed patients, index sensitivity and NPV reached also 100%.

**Conclusion:**

In case of pneumonia suspicion with a CRP level under 130 mg/L (independently of the severity) L-UAT is useless in immunocompetent patients with a NPV at 100%. We must remain cautious in patients with symptoms onset less than 48 h before CRP dosage.

**Supplementary Information:**

The online version contains supplementary material available at 10.1007/s10096-024-04814-x.

## Introduction

Legionnaire’s disease (LD) is a type of pneumonia caused by *Legionella* bacteria, a Gram-negative bacilli, the most common of which is *Legionella pneumophila* (*Lp*) [1]. LD is the predominant form of legionellosis, while Pontiac fever and focal non-pulmonary infections are uncommon [2]. LD represents for 2–9% of community-acquired pneumonia (CAP) cases in United States and Europe [3–5]. *Lp* serogroup 1 is responsible for 85% of LD in United States and Europe [6–8] and for 50% of LD in New-Zealand and Australia [7, 9]. However, involvement of other species of legionellosis in human pathology remain probably underestimated [9, 10]. LD progress usually to a severe pneumonia with a high mortality rate at 5–14% overall cases and 76% in cases treated with inappropriate antibiotics [11].

Surveillance data in reported cases of LD for 31 of the high-income member countries of the Organization for Economic Co-operation and Development (OECD) in 2012 reveals that only 6 countries (including Denmark, France, Italy, New-Zealand, Slovenia and Spain) had a LD prevalence higher than 2 cases per 100 000 inhabitants [3]. In 2021, 2060 cases of LD were identified in France (which represents a rate of 3 per 100 000 inhabitants). In France, 4 in 18 regions had a rate higher than 4 LD per 100 000 inhabitants including *Bourgogne-Franche-Comté* which is the second highest national region’s rate with 4.8 cases per 100 000 inhabitants [12].

*Legionella* Urinary Antigen Test (UAT) identifies LD with a sensitivity of 85% (pooled sensitivity of different UAT methods) for Lp 1 [4, 13–15]. The test is positive within 48–72 h of symptom onset. *Legionella* UAT false-positive are scarce after heating urine [16]; however, UA excretion can remain for several weeks or months after recovery [4, 17]. In Europe, medical guidelines of several countries as England, Spain and Germany recommended systematic *Legionella* UAT in CAP [18–20], while Infectious Diseases Society of America (IDSA) recommended to perform *Legionella* UAT only in cases of severe CAP or in the presence of epidemiological risk factors for LD [21]. French guidelines considering *Legionella* UAT are complex and not updated since 2006; *Legionella* UAT is recommended in cases of (i) hospitalized CAP without microbiological identification; (ii) CAP transferred to Intensive Care Unit (ICU); (iii) outpatients with *Legionella* suspected CAP [22]. Practically, UAT is performed massively in case of suspected CAP, often outside of recommendations in Europe and United States [23, 24] with a low positivity level (for example, a multi-center study in United States [23] observed only 32 positive *Legionella* UAT in 1941 cases of CAP with *Legionella* UAT performed – a rate of 1.6% –).

In case of CAP, biological findings suggestive of LD are hyponatremia, hepatic cytolysis, renal failure and high level of C-reactive protein (CRP) [25–27]. Due to LD severity, a biomarker with a high sensitivity is required to limit false negative cases. A high level of CRP seems to be the most sensitive biomarker for *Legionella* UAT result prediction; thus, we assumed that a low level of CRP predict for negative UAT [26, 27].

We performed this study to determine CRP optimal threshold for *Legionella* UAT negative predictive value (NPV). Secondary objectives are: (i) to confirm that CRP is the most sensitive biomarker among classical *Legionella* biomarkers (especially natremia, alanine aminotransferase (ALT), aspartate aminotransferase (AST) and renal clearance); (ii) to identify situations or population where this cut-off will not be applied; (iii) to estimate the number of tests avoided after application of this cut-off (and savings achieved).

## Materials and methods

### Study population and inclusion criteria

URINELLA was an observational retrospective study in *Nord Franche‐Comté Hospital* (HNFC), France and *Besançon* University Hospital, France. We included all adults (≥ 18 years old) with *Legionella* UAT (negative or positive) performed in the first 72 h of hospital admission through emergency department, during 5 years (from January 1, 2018 to December 31, 2022).

As discussed above, practically, in France, Legionella UAT is mostly performed in case of hospitalized CAP and not only in cases of severe CAP (with ICU admission). To discuss the clinical situation of suspected pneumonia by referring practitioner, we performed an extraction of pneumonia diagnosis using the International Classification of Diseases 10th revision (ICD-10) coding system. This in order to validate CRP threshold in this sub-populations with clinical interest. Hospitalized-acquired pneumonia is defined as pneumonia that occurs at least 48 h following hospitalization [28]; so we excluded from this study patient with *Legionella* UAT performed > 72h after admission and patient who were not admitted at hospital through our emergency department in order to have a homogenous population (especially for biological data extraction). In order to limit the impact of false positive UAT or asymptomatic UA excretion in our study, medical record of each patient with positive UAT was examined by an infectious disease (ID) specialist (TK for *HNFC* patients and SP for *Besançon* Hospital patients). Cases of false positive UAT or asymptomatic UA excretion was confirmed by a second ID specialist (SZ for *HNFC* patients and TK for *Besançon* Hospital patients) on strong criteria: (i) negativity of other Legionella microbiological respiratory samples (PCR and/or culture) and (ii) presence of an alternative confirmed diagnosis. Disagreements were resolved through discussions, and by a third specialist (VG) if required. False positive UAT or asymptomatic UA excretion were excluded (Supplementary Materials).

The aim of URINELLA study was to determine CRP optimal threshold for *Legionella* UAT NPV. BinaxNOW UAT (*Abbott* provider) was performed in all patients. The BinaxNOW UAT is an immunochromatographic test (ICT) screening *Lp* serogroup 1, with a high sensitivity (90%) and specificity (95 to 100%) for LD [13, 29]. Secondary objectives were to explore other predictors of negative *Legionella* UAT (especially classical *Legionella* biomarkers) and validate CRP threshold in sub-populations with clinical interest: patients with pneumonia, severe patients (in case of ICU admission) and immunosuppressed patients. A lower CRP level is expected in immunosuppressed patients comparing to non-immunosuppressed patients. Analyses excluding immunosuppressed patients will be also done (Supplementary Materials). Immunosuppression was defined by the presence of transplantation, cirrhosis, immune deficiency, splenectomy and hematologic malignancies.

We performed this analyze on *HNFC* patients (first population). In order to carry out an external validation of our results from the first population, we performed a second analysis on *Besançon* University Hospital patients (second population).

### Data collection

Clinical and biological data extraction was obtained from medical records. Comorbidities extraction was performed using the ICD-10 coding system. Concerning biological extraction we chose the first biology performed at emergency department admission in order to have a valid comparison.

### Statistical Analysis

Continuous variables were expressed as mean and standard deviation (SD) and compared by Student’s T-test. A *p*-value < 0.05 was considered significant. Categorical variables were expressed as number (%) and compared by Odds Ratio with Confidence Interval (CI). The nonparametric bootstrap method was used to obtain 95% pointwise confidence intervals (95% CI).

Receiver Operating Characteristic-Curve (ROC-Curve) was used with two methods to determine CRP optimal threshold for *Legionella* UAT result prediction: (i) optimal clinical index defined by the highest sensitivity threshold with a specificity which remain ≥ 50% and (ii) classical Youden index. ROC-Curve analyze of others biomarkers will include classical *Legionella* biomarkers (natremia, ALT, AST and renal clearance) and other biomarkers which differed statistically between the comparison patients with *Legionella* positive UAT and patients with *Legionella* negative UAT. All analyses were performed using R v4.2.1.

### Study design and ethics approval

URINELLA study was designed in accordance with the declaration of Helsinki and conducted in accordance with French legislation with approval obtained from the local ethics committee CERUBFC (*Comité d’Ethique pour la Recherche de l’Université de Bourgogne-Franche-Comté*), n°2023–03-09–014. Due to the retrospective nature of the study without human person involvement, the Ethics Committee determined that patient consent was not required.

## Results

### Population Description

**Fig. 1:**
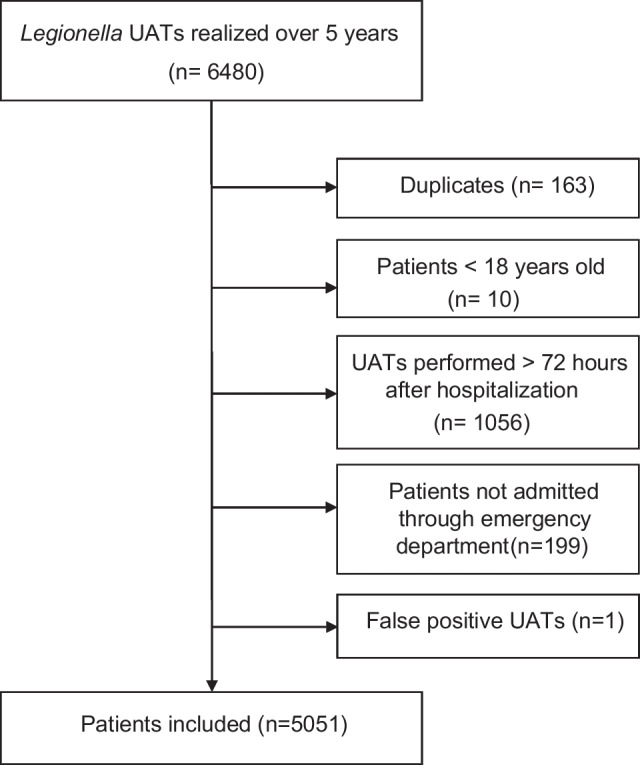
Flow Chart of selection procedure of patients with *Legionella* UAT performed included in URINELLA study (*Nord Franche-Comté* Hospital)

A total of 5051 patients (83 patients with positive *Legionella* UAT and 4968 patients with negative *Legionella* UAT) were included (Fig. 1). The mean age was 72.7 (± 15.0) years with a male predominance (58.4%). Concerning underlying comorbidities, 29.1% had chronic obstructive pulmonary disease (COPD), 11.4% had asthma or bronchiectasis, 10.6% had immunosuppression; 17.6% were smokers. The mean PaO^2^/FiO^2^ ratio was 267.2 ± 122.8 mmHg showing hypoxemia. Laboratory findings revealed a high level of white blood cell count (12.5 ± 7.1 G/l) and high CRP (130.4 ± 110.1 mg/L), a mean of natremia and renal clearance at 137.3 ± 5.4 mmol/L and 70.4 ± 29.2 mL/min, respectively. Concerning outcomes, 23.2% were transferred to ICU with 16.0% of mortality among the 5051 patients.

The 83 patients with positive *Legionella* UAT had pneumonia without any case of Pontiac fever. Patients with positive *Legionella* UAT were younger (respectively 62.6 ± 15.8 years versus 72.9 ± 14.9 years, *p* < 0.001), with a predominance of male (respectively 30% of female versus 42% with an OR 0.60; 95% CI, 0.37–0.95) and smokers (OR 1.60; 95% CI, 0.95–2.60) with less underling diseases than patients with negative *Legionella* UAT (Table 1). Concerning clinical features, patients with positive *Legionella* UAT had a higher fever than patients with negative *Legionella* UAT (respectively 38.9 ± 1.3 °C versus 37.8 ± 1.3 °C, *p* < 0.001), but without any differences regarding clinical presentation’s severity (tachycardia, hypotension and hypoxemia) between the two groups. Concerning classical *Legionella* biomarkers in patients with *Legionella* positive UAT, the mean of natremia was lower (respectively 132.9 ± 4.8 mmol/L versus 137.4 ± 5.3 mmol/L, *p* < 0.001), and CRP was considerably higher (respectively 313.8 ± 113.3 mmol/L versus 127.3 ± 107.4 mmol/L, *p* < 0.001) than patients with negative *Legionella* UAT.

**Table 1:** Characteristics of patients included in URINELLA study and comparison of patients with positive and negative UAT (*Nord Franche-Comté* Hospital patients, n = 5051)

	All patients (N = 5051)^1^	Positive UAT (N = 83)^1^	Negative UAT (N = 4968)^1^	*p*-value^2^	OR (95% CI)
**Demographics data and comorbidities**					
Age (years)	72.7 (15.0)	62.6 (15.8)	72.9 (14.9)	< 0.001	/
Sex (female)	2101 (42%)	25 (30%)	2076 (42%)	/	0.60 (0.37 0.95)
BMI (Kg/m^2^)	28.4 (25.6)	27.0 (5.2)	28.4 (25.9)	0.06	/
Arterial hypertension	2901 (57%)	31 (37%)	2870 (58%)	/	0.44 (0.28 0.68)
Diabetes mellitus	1469 (29%)	18 (22%)	1451 (29%)	/	0.67 (0.39 1.11)
Heart failure	1791 (35%)	17 (20%)	1774 (36%)	/	0.46 (0.26 0.77)
COPD	1468 (29%)	7 (8%)	1461 (29%)	/	0.22 (0.09 0.45)
Asthma or bronchiectasis	578 (11%)	4 (5%)	574 (12%)	/	0.39 (0.12 0.94)
Chronic renal disease	685 (14%)	4 (5%)	681 (14%)	/	0.32 (0.10 0.77)
Malignancy	574 (11%)	4 (5%)	570 (11%)	/	0.39 (0.12 0.94)
Immunosuppression	533 (11%)	4 (5%)	529 (11%)	/	0.42 (0.13 1.03)
Smoking	887 (18%)	21 (25%)	866 (17%)	/	1.60 (0.95 2.60)
Alcohol use disorder	593 (12%)	9 (11%)	584 (12%)	/	0.91 (0.42 1.74)
**Clinical features**					
Temperature (°C)	37.8 (1.3)	38.9 (1.3)	37.8 (1.3)	< 0.001	
> 40 °C	154 (3%)	21 (25%)	133 (3%)	/	12.3 (7.14, 20.5)
MAP (mmHg)	96.6 (27.3)	93.8 (15.5)	96.6 (27.5)	0.11	
< 65 mmHg	218 (4%)	3 (4%)	215 (4%)	/	0.83 (0.20, 2.23)
Heart rate (bpm)	96.9 (22.1)	101.2 (23.0)	96.9 (22.1)	0.093	
> 100 bpm	2118 (42%)	41 (50%)	2077 (42%)	/	1.38 (0.89, 2.13)
PaO2/FiO_2_ (mmHg)	267.2 (122.8)	268.8 (87.5)	267.2 (123.3)	0.9	
< 300 mmHg	2842 (56%)	46 (55%)	2796 (56%)	/	0.97 (0.63, 1.50)
< 200 mmHg	1094 (22%)	11 (13%)	1083 (22%)	/	0.55 (0.27, 0.99)
< 100 mmHg	216 (4%)	2 (2%)	214 (4%)	/	0.55 (0.09, 1.75)
**Laboratory findings**					
Leukocytes (G/l)	12.5 (7.1)	13.7 (10.5)	12.5 (7.1)	0.3	/
Neutrophils (G/l)	10.1 (5.7)	10.8 (3.5)	10.1 (5.7)	0.07	/
Eosinophils (G/l)	0.1 (0.2)	0.0 (0.0)	0.1 (0.2)	< 0.001	/
Basophils (G/l)	0.0 (0.1)	0.0 (0.0)	0.0 (0.1)	0.11	/
Lymphocytes (G/l)	1.3 (3.0)	2.0 (10.4)	1.3 (2.7)	0.5	/
Monocytes (G/l)	0.9 (1.1)	0.7 (0.4)	0.9 (1.1)	< 0.001	/
Hemoglobin (g/dL)	12.8 (2.2)	13.5 (1.7)	12.8 (2.2)	< 0.001	/
Platelets (G/l)	259.1 (121.4)	236.2 (83.8)	259.5 (121.9)	0.02	/
Sodium (mmol/L)	137.3 (5.4)	132.9 (4.8)	137.4 (5.3)	< 0.001	/
< 130 mmol/L	300 (6%)	15 (18%)	285 (6%)	/	3.62 (1.97, 6.23)
Potassium (mmol/L)	4.1 (0.6)	3.9 (0.5)	4.1 (0.6)	< 0.001	/
Urea (mmol/L)	9.2 (6.8)	8.8 (5.6)	9.2 (6.8)	0.5	/
Renal clearance (mL/min)	70.4 (29.2)	72.0 (28.0)	70.4 (29.3)	0.6	/
< 60 mL/min	1808 (36%)	27 (33%)	1781 (36%)	/	0.86 (0.53, 1.36)
AST (U/l)	68.6 (253.9)	158.8 (707.0)	67.0 (238.3)	0.3	/
ALT (U/l)	49.3 (160.1)	124.4 (665.4)	47.9 (134.3)	0.3	/
> 70 U/l	497 (11%)	19 (24%)	478 (11%)	/	2.56 (1.48, 4.25)
ALP (U/l)	108.7 (121.2)	106.1 (95.1)	108.7 (121.7)	0.8	/
Gamma-GT (U/l)	90.6 (155.7)	83.9 (104.7)	90.7 (156.5)	0.6	/
CRP (mg/L)	130.4 (110.1)	313.8 (113.3)	127.3 (107.4)	< 0.001	/
> 200 mg/L	1111 (22%)	66 (80%)	1045 (21%)	/	14.6 (8.72, 25.7)
> 300 mg/L	444 (9%)	44 (53%)	400 (8%)	/	12.8 (8.23, 20.0)
Fibrinogen (g/L)	5.8 (2.1)	8.1 (2.2)	5.8 (2.1)	< 0.001	/
Prothrombin (%)	77.3 (24.3)	78.8 (23.8)	77.3 (24.3)	0.6	/
Lactate (mmol/L)	1.9 (1.8)	2.1 (2.0)	1.9 (1.8)	0.5	/
**Outcome**					
Hospitalization (days)	13.5 (14.3)	10.8 (9.7)	13.6 (14.4)	0.01	/
ICU admission	1172 (23%)	25 (30%)	1147 (23%)	/	1.44 (0.88, 2.28)
IMV	374 (7%)	10 (12%)	364 (7%)	/	1.73 (0.83, 3.23)
Death	805 (16%)	8 (10%)	797 (16%)	/	0.56 (0.25, 1.09)

^1^ Mean (SD); n (%)

^2^ Welch Two Sample t-test

*UAT * urinary antigen test, *OR *odds ratio, *CI*  confidence interval, *BMI *body mass index, *COPD *chronic obstructive pulmonary disease, *MAP *mean arterial pressure, *bpm *beats per minute, *PaO2 *arterial oxygen pressure, *FiO2 *fractional inspired oxygen, *AST *aspartate aminotransferase, *ALT *alanine aminotransferase, *ALP*, alkaline phosphatase, *ICU *intensive care unit, *IMV *invasive mechanical ventilation

### CRP optimal threshold determination and sensitivity analysis

**Fig. 2:**
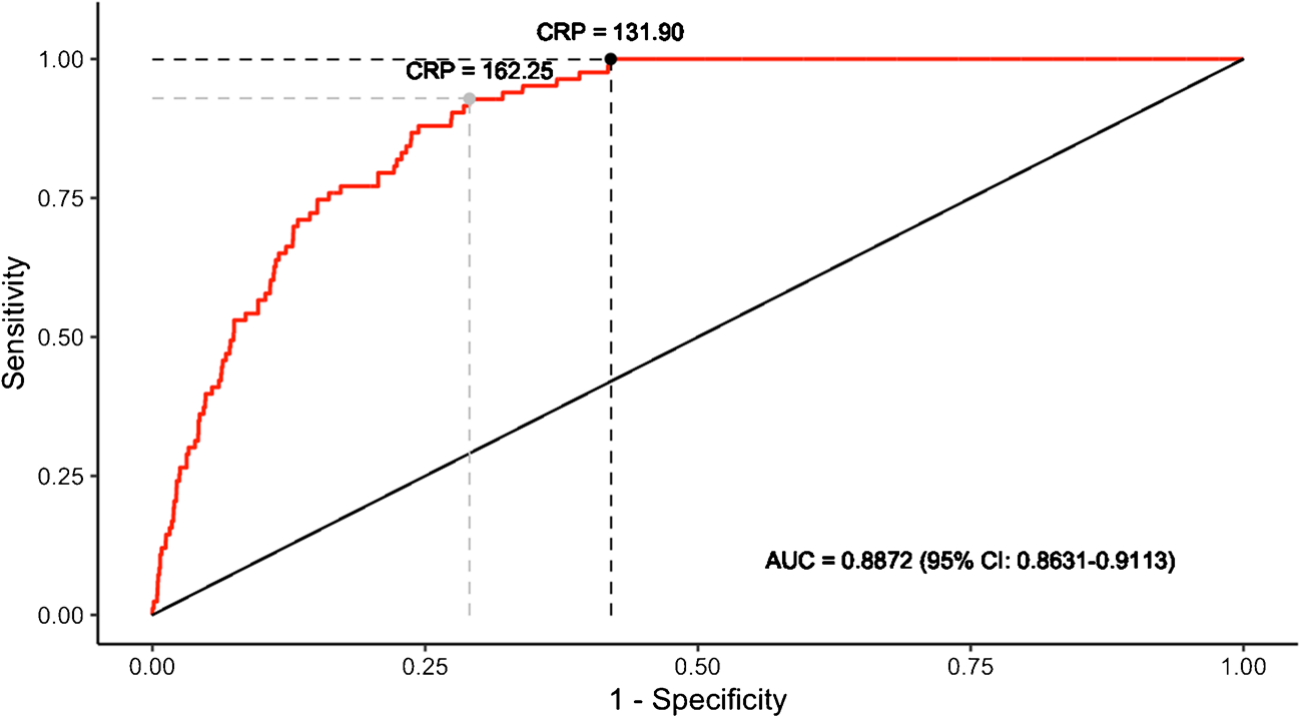
Receiver Operating Characteristic-Curve for positive *Legionella* UAT based on C-Reactive-Protein level in *Nord Franche-Comté* Hospital patients (n = 5051). Dark and grey points represent optimal clinical index and classical Youden index, respectively

CRP optimal clinical index (the highest sensitivity with a specificity which remains ≥ 50%) was 131.9 mg/L, with a negative predictive value (NPV) at 100%, sensitivity at 100% and specificity at 58.0%. Using the Youden’s method CRP threshold for *Legionella* UAT result prediction was 162.25 mg/L (Fig. 2) with a NPV at 99.8%, sensitivity at 92.9% and specificity at 70.9%.

**Fig. 3:**
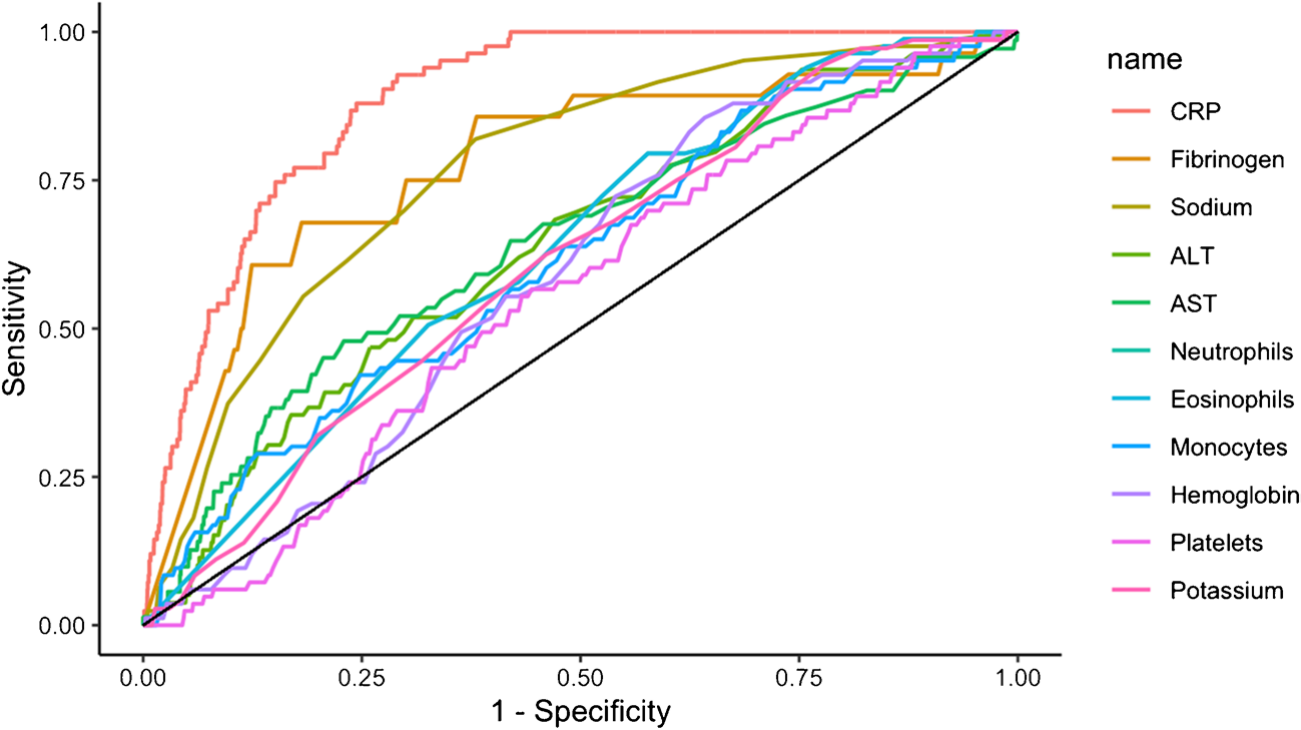
Receiver Operating Characteristic-Curve for positive *Legionella* UAT based on selected biomarkers levels in *Nord Franche-Comté* Hospital patients (n = 5051)

**Fig. 4:**
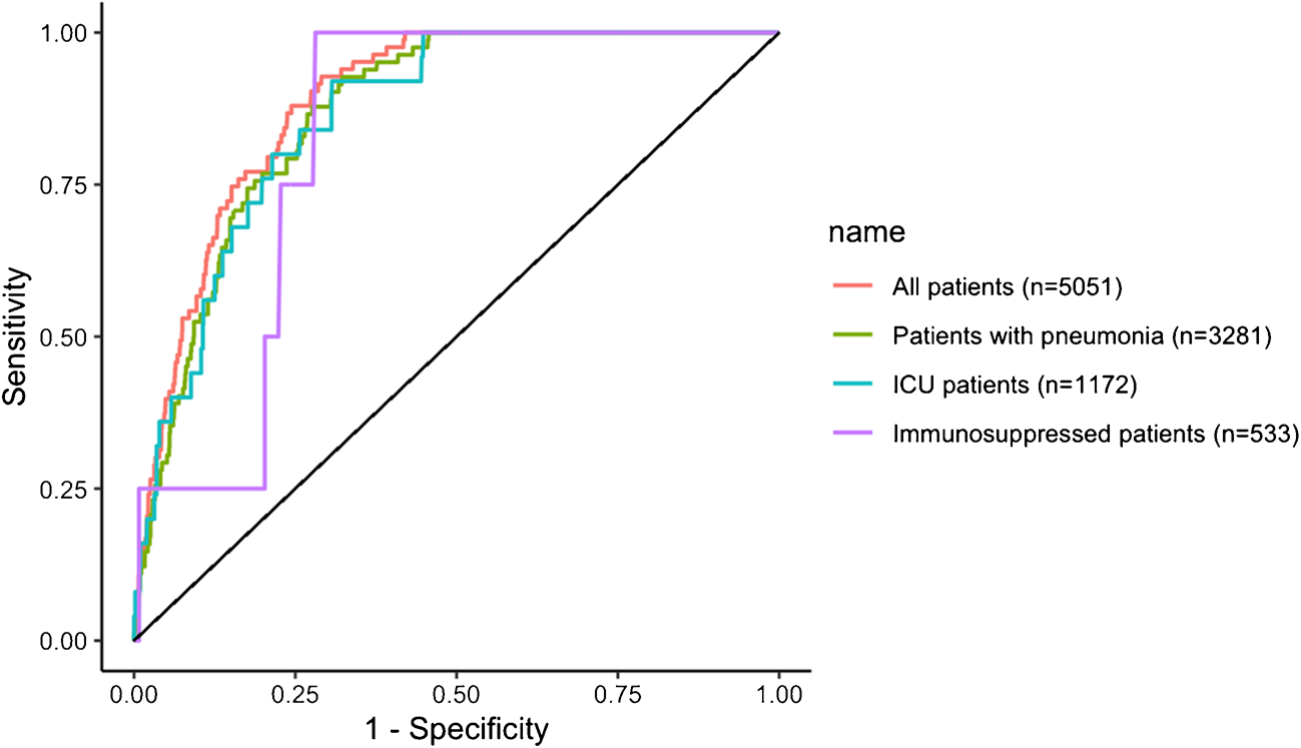
Receiver Operating Characteristic-Curve for positive *Legionella* UAT based on C-Reactive-Protein level for different clinical status in *Nord Franche-Comté* Hospital patients (n = 5051)

Considering other laboratory findings (classical *Legionella* biomarkers and other biomarkers which differed statistically between patients with positive *Legionella* UAT and patients with negative *Legionella* UAT), the Area Under the Curve (AUC) of the different ROC-Curve confirmed that CRP is the optimal biomarker for *Legionella* UAT result prediction (Fig. 3) with an AUC at 88.7% (95% CI, 86.3–91.1). ROC-Curve aspect of the different sub-populations confirmed that these CRP levels can be used in patients with pneumonia (AUC 86.8%; 95% CI, 84.1–89.5) and severe patients (AUC 86.4%; 95% CI, 81.1–91.7) but we must remain cautious in immunosuppressed patients (AUC 82.1%; 95% CI, 70.2–94.0) (Fig. 4).

Analyses excluding immunosuppressed patients and including False positive UAT are presented in Supplementary Materials (Annex 2 and Annex 1 respectively).

### Cost saving by applying a cut-off of CRP level at 130 mg/L

Among the *HNFC* patients, 56.4% (2849/5051) had a CRP level under 130 mg/L and 57.6% (2427/4216) among *Besançon* Hospital patients. BinaxNOW UAT costs 11.34€ at HNFC and 12€ at Besançon Hospital.

Applying a cut-off of CRP level at 130 mg/L (no *Legionella* UAT if CRP under 130 mg/L) it would safe 32307.66€ at HNFC and 29124€ at Besançon Hospital during the study period.

### External validation on a second population

A total of 4216 patients (42 patients with positive *Legionella* UAT and 4174 patients with *Legionella* negative UAT) were included (Annex 3). The mean age was 70.4 (± 15.8) years with a male predominance (59.7%) with similar comorbidities to *HNFC* patients (Annex 4).

**Fig. 5:**
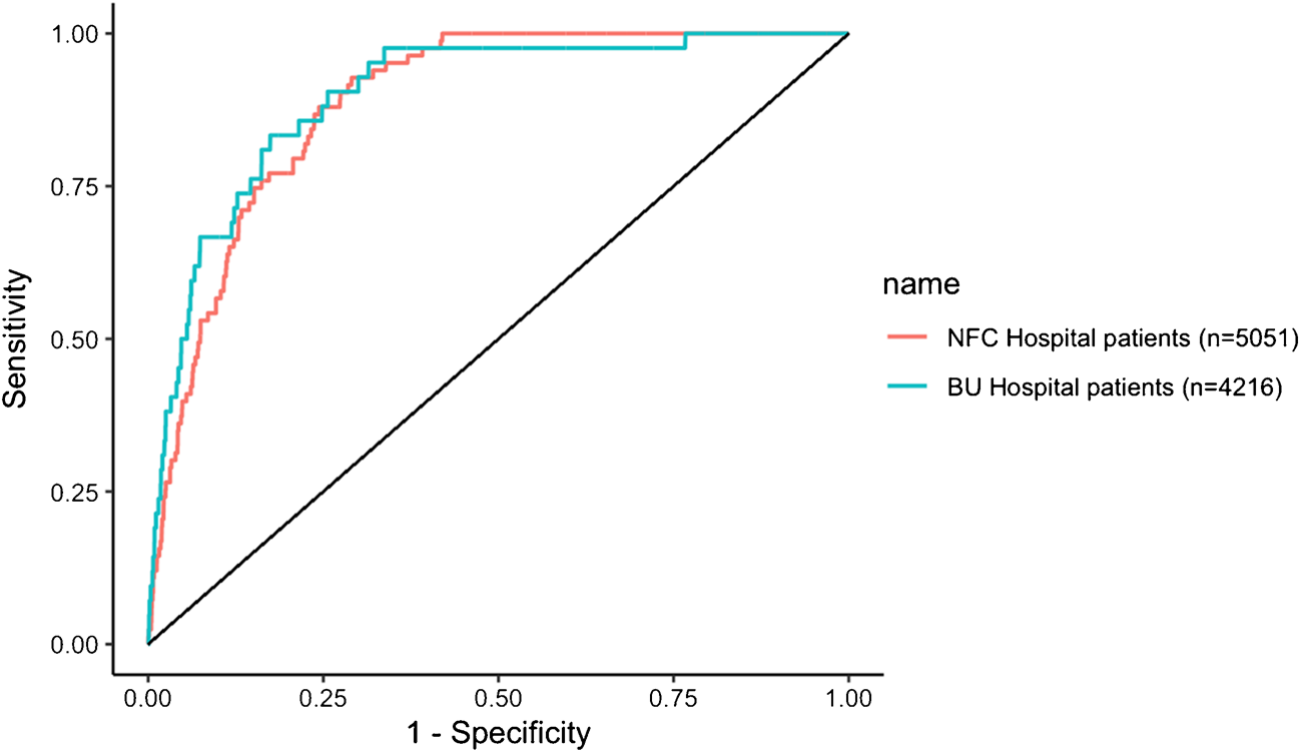
Receiver Operating Characteristic-Curve for positive *Legionella* UAT based on C-Reactive-Protein level in *Nord Franche-Comté* (NFC) Hospital patients (n = 5051) and in *Besançon* University (BU) Hospital patients (n = 4216)

The analysis of *Besançon* University Hospital patients confirms our results with similar characteristics for a CRP threshold at 131.9 mg/L with a NPV at 99.9%, sensitivity at 97.6% and specificity at 59.1%; and a similar ROC-curve with an AUC at 89.8% (95% CI, 85.5–94.1) (Fig. 5).

For information, in *Besançon* University Hospital patients optimal clinical index reached 161.05 mg/L, with a NPV at 99.9%, sensitivity at 97.6% and specificity at 66.3%; Youden index at 245.45 mg/L with a NPV = 99.8%, sensitivity at 83.3% and specificity at 82.6%) (Annex 5).

The ROC-Curve of the different sub-populations of *Besançon* University Hospital patients confirmed that CRP levels can be also used in patients with pneumonia (AUC 82.3%; 95% CI, 72.0–92.7) and critical patients (AUC 93.0%; 95% CI, 87.1–99.0). However, we cannot validate these results with immunosuppressed patients (AUC 70.0%; 95% CI, 20.2–100) (Annex 6); moreover if we exclude this population, optimal clinical index sensitivity reach at 100%, as in the first population (*HNFC* patients) (Annex 7).

## Discussion

URINELLA study (i) determines that CRP optimal threshold for *Legionella* UAT NPV could be approximately 130 mg/L with a high sensitivity; (ii) confirms that CRP is the most sensitive biomarker among classical *Legionella* biomarkers; (iii) identifies that this cut-off may not be applied in immunosuppressed patients and (iv) demonstrates cost saving by applying this cut-off.

To our knowledge no studies focuses specifically in CRP as a predictor of negative *Legionella* UAT, in medical literature. Only one study (*Roed* et al.) explored the predictors of positive or negative *Legionella* UAT in CAP [26], in comparison to several studies for pneumococcal UAT [30]. Thus, *Roed* et al. [26] in a cohort of 100 pneumonia (25 positive *Legionella* UAT compared to 75 negative *Legionella* UAT) showed that a CRP level > 200 mg/L had a sensitivity at 92% in the group positive *Legionella* UAT *versus* only 27% in the group negative *Legionella* UAT). However, the study was not build to determine a CRP threshold for *Legionella* UAT NPV. Comparatively in our study, a CRP level > 200 mg/L had a sensitivity at 80% in the group positive Legionella UAT versus 21% in the group negative Legionella UAT.

No one of the 1770 (35%) patients without pneumonia had a positive Legionella UAT test, which emphasize that *Legionella* UAT should perform only in case of pneumonia. URINELLA study included all adult inpatients with *Legionella* UAT performed in the first 72 h of hospital admission through emergency department. In France, *Legionella* UAT is recommended in hospitalized patients with CAP without microbiological identification or in patients with CAP transferred to ICU [22]. Practically, UAT is performed massively, often outside of recommendations and possibly outside of CAP diagnosed [24]. Our population highlighted this practice with only 3281/5052 (64.9%) patients with confirmed pneumonia diagnosis. In our study, patients with positive *Legionella* UAT (n = 83) had a male predominance (69%), a mean age at 62.6 ± 15.7 years and 25% of smokers. *Allgaier *et al*.,* in a cohort of 177 United States hospitals with 642 Legionellosis with positive UAT found also a male predominance (62.9%), a median age at 62.0 (52.0–73.0) years and 38% of smokers [31]. Regarding comorbidities, patients with positive *Legionella* UAT had less underling diseases than patients with *Legionella* negative UAT. This could be explained by: (i) a selected control population of hospitalized patients with comorbidities; (ii) a lower mean age in the positive *Legionella* UAT group than in the negative *Legionella* UAT group with less expected comorbidities in younger patients. In our study, the referring practitioner has done *Legionella* UAT in case of *Legionella* suspicion. Therefore, a high level of CRP without no anamnestic and/or clinical and/or biological signs in favor of Legionella CAP suspicion should not lead to Legionella UAT.

In case of pneumonia, LD is not systematically considered in empirical regimen of antibiotics, especially in mild-to-moderate pneumonia; however LD could progress to a severe pneumonia with a high rate of mortality. Due to LD severity, a biomarker with a high sensitivity is required to limit false negative cases. In our study, a CRP threshold at 131.9 mg/L had 100% of sensitivity and can predict for a negative *Legionella* UAT in 100% of cases in our population; however the wide 95% CI of AUC observed in immunosuppressed patients ROC-curve do not allow us to support our conclusions in this sub-population. If we exclude immunosuppressed patients in *Besançon* University Hospital population a CRP threshold at 161.05 mg/L reach also 100% of sensitivity which confirmed our first analysis.

The prevalence of the disease is low (1.6%, n = 83/5051), this low prevalence is associated with a higher value of the negative predictive value of Legionella UAT. However, by definition sensitivity is not affected by a low disease prevalence, and in the present study sensitivity remains at 100%, which confirm the strong relevance of a CRP threshold at 130 mg/L. One of our limitations could be the selection of the study population with the exclusion of false positives *Legionella* UAT, however two ID specialists must be in agreement in this case and the analysis of the whole population (which include false positives *Legionella* UAT) hasn’t change CRP thresholds (Appendix 1). Concerning the study population, a third of patients had *Legionella* UAT performed without pneumonia diagnosis; the extraction was based on patients with a ICD-10 diagnosis of pneumonia and it could miss some pneumonia diagnostic due to coding (however this would not negate the assumption of a misused of Legionella UAT, performed outside of CAP); furthermore, we made the analyses in this sub-population which was conclusive. BinaxNOW urinary antigen test used for *Legionella* UAT has a sensitivity of 90% and screens almost only Lp serogroup 1; so, the diagnosis of LD shall not rule out especially LD due to other Lp serogroup than serogroup 1. Elevation of CRP biomarker could take 24–48 h in case of infection [32] and we have no explored the number of patients in this situation in our study. For these reasons URINELLA do not conclude that a CRP under 130 mg/L rule out definitely the diagnosis of LD; this result concerns only serotype 1 *Legionella* infections. However, our study highlight the futility of *Legionella* UAT dosage in these cases.

In case of pneumonia (including severe pneumonia) *Legionella* UAT seems to be useless when CRP is under 130 mg/L in immunocompetent patients with a NPV at 100%. However, these results need to be confirmed in patients with symptoms onset less than 24–48 h before CRP dosage.

### Supplementary Information

Below is the link to the electronic supplementary material.

Supplementary file1 (DOCX 551 KB)

## Data Availability

Data are available on request from the corresponding author due to privacy restrictions.
