# Supplementary material for: CRP under 130 mg/L rules out the diagnosis of Legionella pneumophila serogroup 1 (URINELLA Study)
Source: Eur J Clin Microbiol Infect Dis. 2024 Mar 26;43(6):1051–9. doi: 10.1007/s10096-024-04814-x (PMC11178638; doi:10.1007/s10096-024-04814-x)
Supplement: Supplementary file 1 — Supplementary file1 (DOCX 551 KB) [file 10096_2024_4814_MOESM1_ESM.docx]

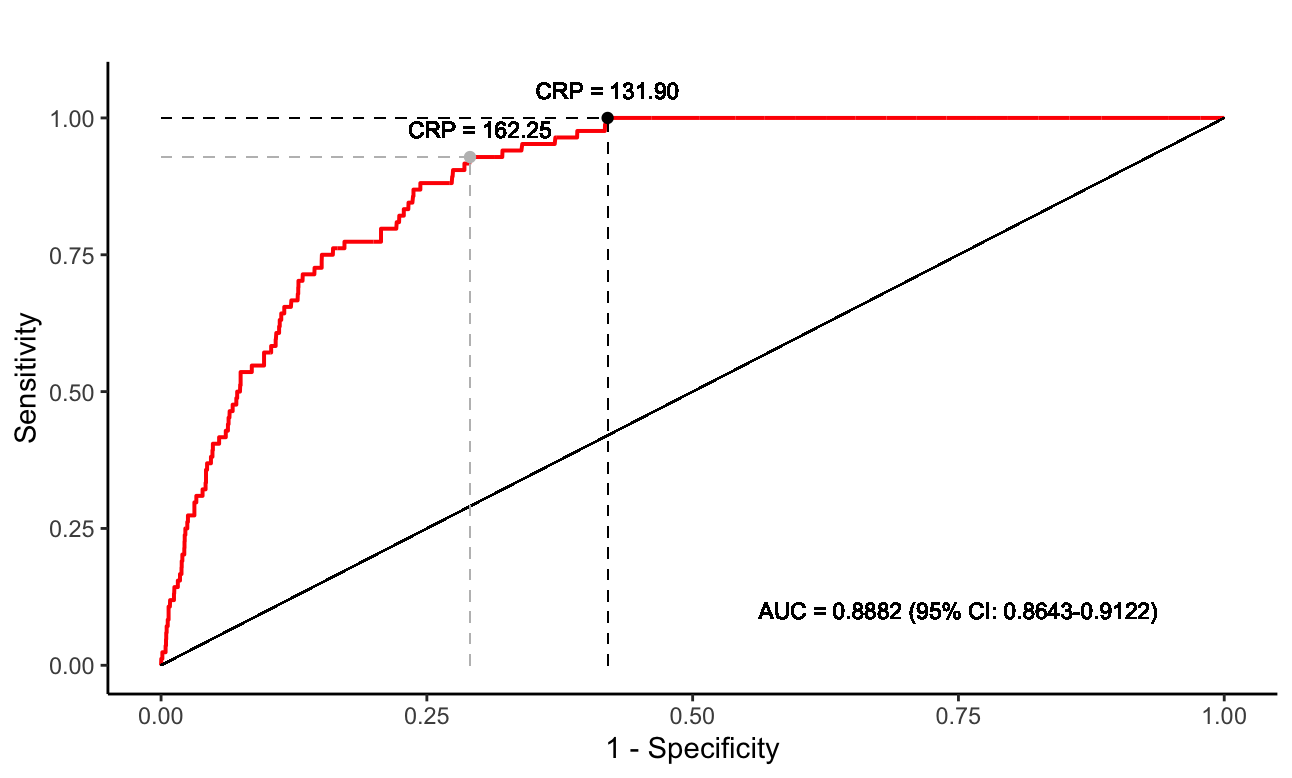
**Supplementary Materials (Annex 1 to 7)**


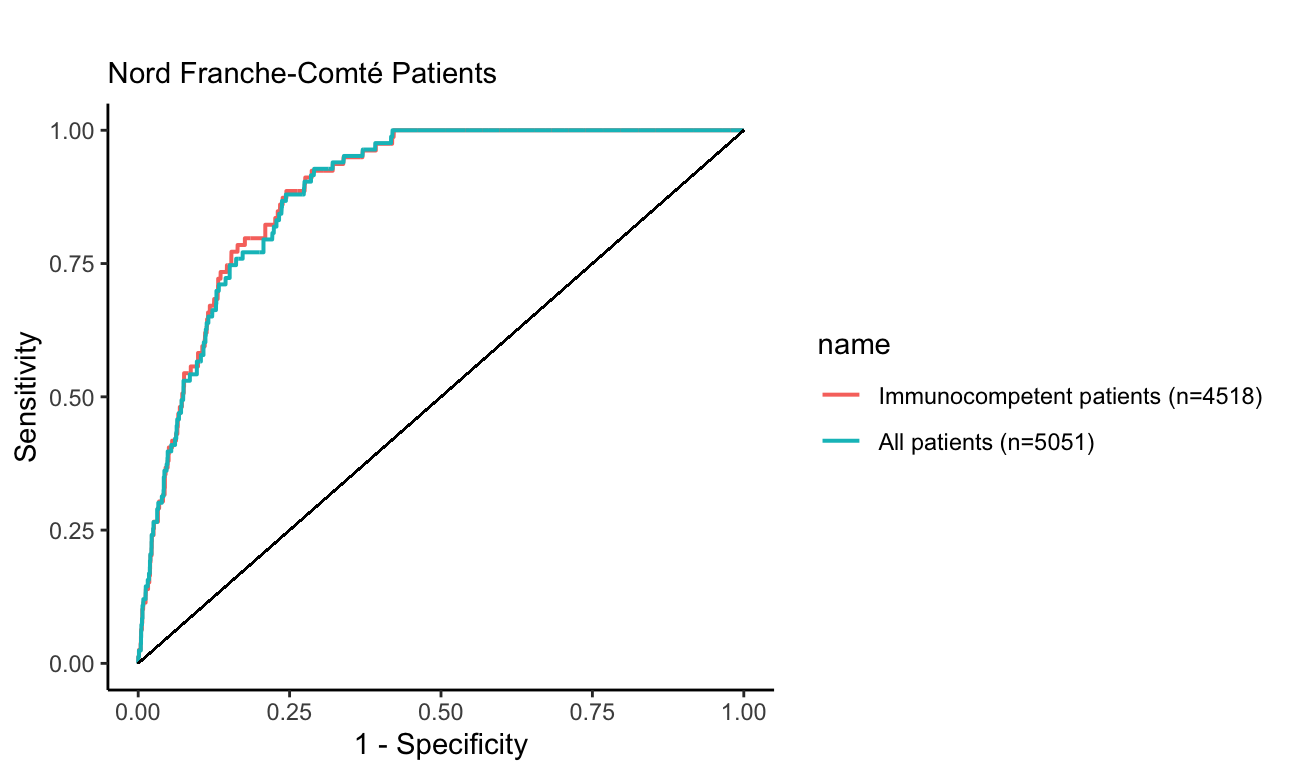
**Annex 1.** Receiver Operating Characteristic-Curve for positive *Legionella* UAT based on C-Reactive-Protein level including false positive UAT or asymptomatic UA excretion in *Nord Franche-Comté* Hospital patients (n=5052).

**Annex 2.** Receiver Operating Characteristic-Curve for positive *Legionella* UAT based on C-Reactive-Protein level in immunocompetent patients (n=4518) and in all patients (n=5051) in *Nord-Franche-Comté* Hospital.

*Legionella* UATs realized over 5 years (2018-2022) (n=6024)

Patients included (n= 4216)

Duplicates (n= 18)

UATs performed > 72 hours after hospitalization (n=1133)

Patients not admitted through emergency department (n=631)

Patients < 18 years old (n=23)

False Positive UATs (n=3)

**Annex 3.** Flow Chart of selection procedure of patients with *Legionella* UAT performed included in URINELLA Study (*Besançon* University Hospital).

|  | ***Besançon Hospital* patients^1^**  (N = 4 216) | ***HNFC* patients^1^**  (N = 5 051) |
| --- | --- | --- |
| Age (years) | 70.4 (15.8) | 72.7 (15.0) |
| Sex (female) | 1668 (40%) | 2101 (42%) |
| Arterial hypertension | 2602 (62%) | 2901 (57%) |
| Diabetes mellitus | 1268 (30%) | 1469 (29%) |
| Heart failure | 1476 (35%) | 1791 (35%) |
| COPD | 1150 (27%) | 1468 (29%) |
| Asthma or bronchiectasis | 349 (8%) | 578 (11%) |
| Chronic kidney disease | 342 (8%) | 685 (14%) |
| Malignant tumor | 575 (14%) | 574 (11%) |
| Immunosuppression | 536 (13%) | 533 (11%) |
| Smoking | 1471 (35%) | 887 (18%) |
| Alcohol use disorder | 497 (12%) | 593 (12%) |
| CRP (mg/L) | 135.1 (115.1) | 130.4 (110.1) |
| ICU admission | 1024 (24%) | 1172 (23%) |
| IMV | 633 (15%) | 374 (7%) |
| Death | 576 (15%) | 805 (16%) |
| ^1^ Mean (SD); n (%)  Abbreviations: COPD = Chronic Obstructive Pulmonary Disease,  CRP: C-reactive protein ICU = Intensive Care Unit, IMV = Invasive Mechanical Ventilation | | |

**Annex 4.** Characteristics of patients included in URINELLA study in Besançon University Hospital (n=4216) and *Nord Franche-Comté* Hospital (n=5051).


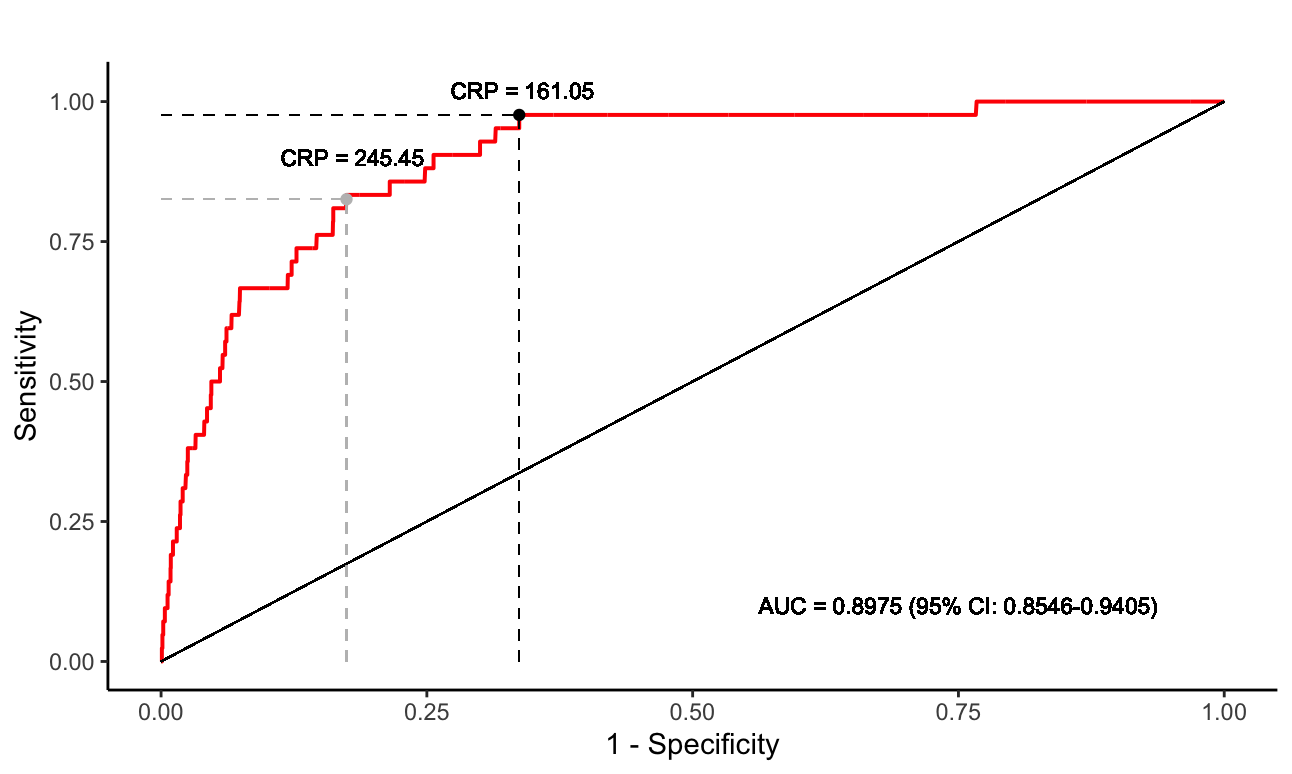
**Annex 5.** Receiver Operating Characteristic-Curve for positive *Legionella* UAT based on C-Reactive-Protein level in *Besançon* University patients (n=4216). Dark and grey points represent optimal clinical index and classical Youden index, respectively.


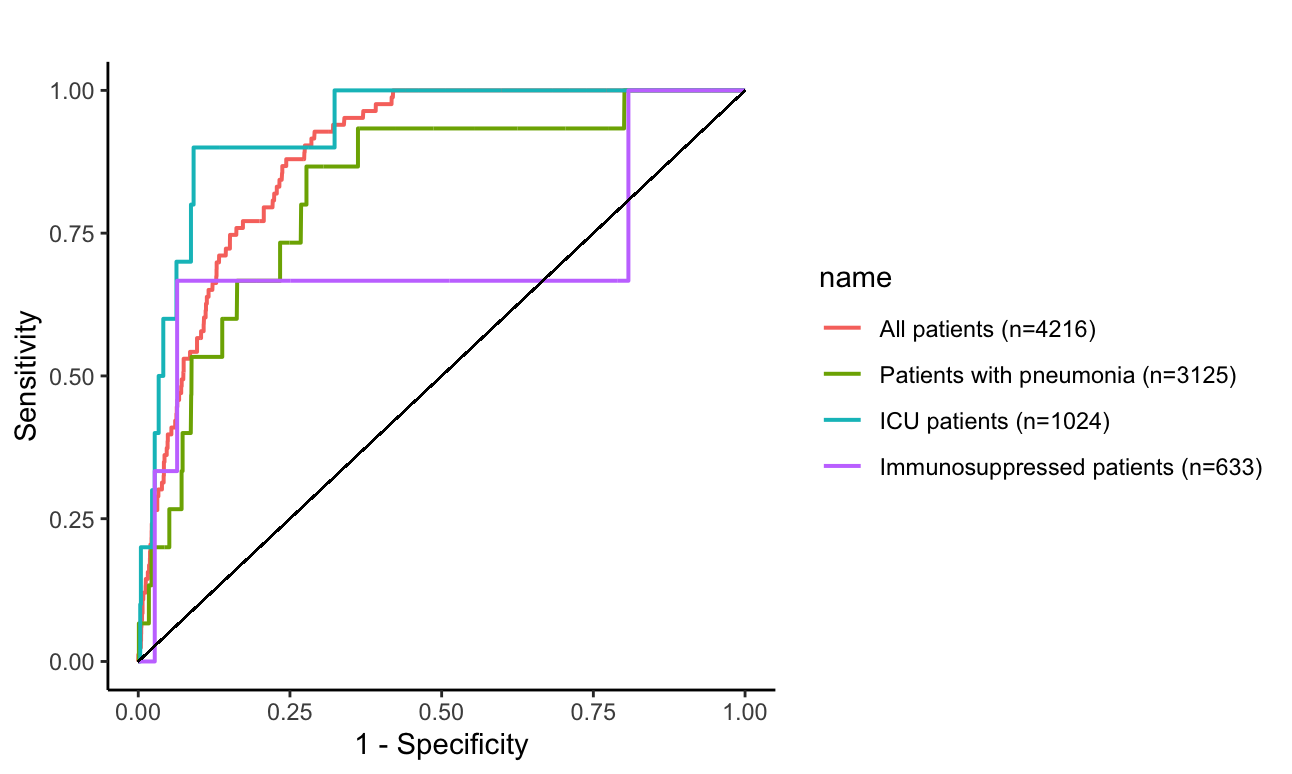


**Annex 6.** Receiver Operating Characteristic-Curve for positive *Legionella* UAT based on C-Reactive-Protein level for different clinical status in *Besançon* University Hospital patients (n=4216).


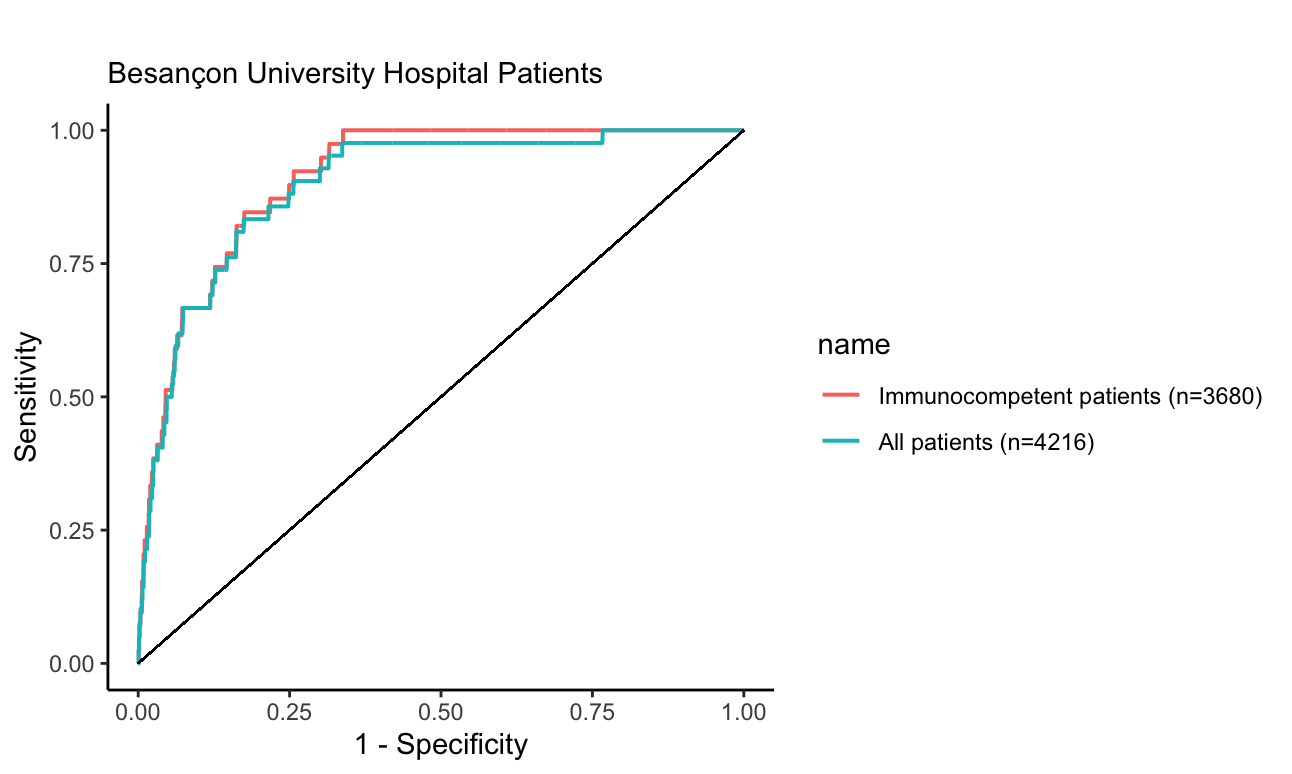
**Annex 7.** Receiver Operating Characteristic-Curve for positive *Legionella* UAT based on C-Reactive-Protein level in immunocompetent patients (n=3680) and in all patients (n=4216) in *Nord-Franche-Comté* Hospital.
